# Effectiveness of mRNA-1273, BNT162b2, and JNJ-78436735 COVID-19 Vaccines Among US Military Personnel Before and During the Predominance of the Delta Variant

**DOI:** 10.1001/jamanetworkopen.2022.8071

**Published:** 2022-04-20

**Authors:** Angelia A. Eick-Cost, Saixia Ying, Natalie Wells

**Affiliations:** 1 Armed Forces Health Surveillance Division, Defense Health Agency, Silver Spring, Maryland

## Abstract

**Importance:**

No studies to date have evaluated the effectiveness of 3 COVID-19 vaccines in the US military population, especially during the circulation of the SARS-CoV-2 Delta (B.1.617.2) variant.

**Objective:**

To estimate the effectiveness of the mRNA-1273, BNT162b2, and JNJ-78436735 vaccines among US military personnel before and during the predominance of the Delta variant in the US.

**Design, Setting, and Participants:**

This case-control study was conducted among all unvaccinated and fully vaccinated US military personnel who had a documented SARS-CoV-2 test performed in the US between January 1 and September 24, 2021. Individuals were identified using Department of Defense (DOD) electronic medical, laboratory, and surveillance databases. The pre-Delta period was defined as January 1 to May 31, 2021, and the Delta period as June 19 to September 24, 2021. Case individuals were defined by a positive polymerase chain reaction SARS-CoV-2 test result or a positive antigen test result with symptoms. Control individuals had at least 1 negative SARS-CoV-2 test result.

**Exposures:**

COVID-19 vaccination with the mRNA-1273, BNT162b2, or JNJ-78436735 vaccine, assessed from DOD electronic vaccination records.

**Main Outcomes and Measures:**

COVID-19 vaccine effectiveness overall, by vaccine type, and by outcome stratified by the pre-Delta and Delta periods in the US. Vaccine effectiveness was estimated as 100 × (1 − odds ratio) in a logistic regression model with adjustment for potential confounders.

**Results:**

The cohort included 441 379 individuals, with 290 256 in the pre-Delta period (236 555 [81%] male; median age, 25 years [range, 17-68 years]) and 151 123 in the Delta period (120 536 [80%] male; median age, 26 years [range, 17-70 years]). Adjusted vaccine effectiveness of all vaccines was significantly higher during the pre-Delta period (89.2%; 95% CI, 88.1%-90.1%) compared with the Delta period (70.2%; 95% CI, 69.3%-71.1%) for all outcomes, an overall decrease of 19%. mRNA-1273 vaccine effectiveness was highest in the pre-Delta (93.5%; 95% CI, 91.9%-94.7%) and Delta (79.4%; 95% CI, 78.3%-80.4%) periods for all outcomes, whereas the JNJ-78436735 vaccine had the lowest effectiveness during the pre-Delta (81.8%; 95% CI, 74.2%- 87.1%) and Delta (38.3%; 95% CI, 34.5%-41.9%) periods. Effectiveness for all vaccines during both periods was higher for symptomatic infection and hospitalization among individuals with SARS-CoV-2 infection.

**Conclusions and Relevance:**

In this case-control study, among US military personnel, COVID-19 vaccine effectiveness was significantly lower during the period when the Delta variant predominated compared with the period before Delta variant predominance; this was especially true for the JNJ-78436735 vaccine. These findings were confounded by time since vaccination; this and the change in effectiveness support the need for booster doses and continued evaluation of vaccine effectiveness as new variants of SARS-CoV-2 emerge.

## Key Points

**Question:**

Did the effectiveness of the mRNA-1273, BNT162b2, and JNJ-78436735 COVID-19 vaccines change among US-based military personnel before and during the predominance of the SARS-CoV-2 Delta (B.1.617.2) variant?

**Findings:**

In this case-control study of 441 379 active US military personnel, overall COVID-19 vaccine effectiveness decreased by 19% from the pre-Delta to the Delta period. JNJ-78436735 had the lowest overall vaccine effectiveness in the pre-Delta (81.8%) and Delta (38.3%) periods.

**Meaning:**

In this study, COVID-19 vaccine effectiveness decreased among US-based military personnel during the time of SARS-CoV-2 Delta variant predominance, especially for recipients of the JNJ-78436735 vaccine; this finding supports the use of booster doses to increase effectiveness.

## Teaser

This case-control study evaluates the effectiveness of 3 COVID-19 vaccines for infection and hospitalization outcomes among US military personnel before and during the predominance of the Delta variant in the US.

## Introduction

Randomized clinical trials have shown that the current mRNA-based COVID-19 vaccines have high efficacy (94%-95%) for preventing COVID-19.^1,2^ The adenovirus-vectored vaccine, JNJ-78436735, was also found to have moderately high efficacy against symptomatic COVID-19 (66%) and high efficacy against hospitalization for COVID-19 (93%).^3,4^ In an evaluation of the vaccines, several effectiveness studies have been conducted on mRNA COVID-19 vaccines. Most focused on hospital-based health care workers or frontline workers and the elderly population, and only a few assessed vaccine effectiveness against the Delta variant.^5,6,7,8,9,10,11,12,13,14,15,16,17,18,19^ There is also a paucity of data on the effectiveness of the JNJ-78436735 vaccine.^19,20,21,22^

A critical population that has not been adequately captured in civilian-based studies of vaccine effectiveness is US military personnel, who differ from the general population by age, health status, access to medical care, and potential for greater SARS-CoV-2 exposure. This population can be considered at high risk of exposure to SARS-CoV-2 given their worldwide deployment, close-quarter living and working conditions, and participation in humanitarian and public health logistical operations such as COVID-19 mass vaccination sites. The Department of Defense (DOD) began administering COVID-19 vaccines to military personnel in mid-December 2020. The DOD followed guidance from the Centers for Disease Control and Prevention on prioritization of COVID-19 vaccines, and initial distribution sites were at major military treatment facilities.^23^ However, military personnel could also receive a vaccine at any non-DOD location, and vaccine type was an individual decision. Mandatory vaccine requirements for all military personnel did not occur until August 24, 2021.^24^ After SARS-CoV-2 testing assays were available, the DOD conducted testing of symptomatic and asymptomatic exposed military personnel; screening tests before deployment, redeployment, and training; routine surveillance testing; and pre- and posttravel testing in accordance with federal guidelines.^25^ To date, there have been only 2 published articles^26,27^ on vaccine effectiveness restricted to US veterans and not to currently serving military personnel. Thus, this study evaluated the effectiveness of 3 COVID-19 vaccines administered to serving military personnel before and during the predominance of the Delta (B.1.617.2) variant to provide better evidence for policy recommendations to maintain a medically ready military force.

## Methods

### Study Population

In this case-control study, the study population consisted of all active component, US-based military personnel with a documented SARS-CoV-2 laboratory test conducted in the US between January 1 and September 24, 2021. The population was identified using DOD electronic laboratory data, specifically from the Composite Health Care System and the Military Health System’s MHS GENESIS laboratory data, which were standardized and provided by Navy public health officials. Military personnel from the Reserves or National Guard and those who had a positive SARS-CoV-2 laboratory test result before January 1, 2021, were excluded. This study followed the Strengthening the Reporting of Observational Studies in Epidemiology (STROBE) reporting guideline. The study was reviewed by the DOD Office for Human Research Protections at the Defense Health Agency Office of Research Protections and was determined to not constitute human participants research; thus, the need for informed consent was waived.

### Study Design

A case-control design was used to evaluate the effectiveness of COVID-19 vaccines. Case individuals were defined as those with a positive polymerase chain reaction SARS-CoV-2 test result (83.7% of cases) or a positive result of SARS-CoV-2 antigen and/or unknown test type with no polymerase chain reaction test conducted and a medical encounter within 10 days before or after the test collection date with a COVID-like illness diagnosis (16.3% of cases) (eAppendix in the Supplement). The Defense Medical Surveillance System,^28^ a longitudinal database with near-complete capture of military personnel’s medical encounters, was used to identify medical encounters for COVID-like illness. The earliest case-defining specimen collection date was selected. Control individuals had 1 or more negative SARS-CoV-2 laboratory test result (polymerase chain reaction, antigen, and/or unknown test type). If multiple specimens tested negative, random selection was used to select 1 negative specimen per control individual. The reason for SARS-CoV-2 testing was not considered when selecting participants.

The study period was stratified into pre-Delta and Delta periods. Stratification was based on when the SARS-CoV-2 Delta variant became the predominant strain in the US.^29^ The pre-Delta period was defined as January 1 to May 31, 2021, and the Delta period was defined as June 19 to September 24, 2021 (end of data collection). This stratification was used because identifiable SARS-CoV-2 sequencing data were not available. Participants with a test collection date from June 1 to 18, 2021, were excluded from the study because this was considered a washout period when multiple variants were circulating in the US. Demographic, personnel, location, medical, and vaccination data were obtained from the Defense Medical Surveillance System. Additional symptom and hospitalization data were obtained from the Disease Reporting System internet (DRSi),^30^ the official repository for reportable medical events within the DOD.

### Vaccination Status

Vaccine data came from the Defense Enrollment Eligibility Reporting System, which receives data from each service branch’s electronic vaccine tracking system. Vaccination status was determined at the specimen collection date. Participants received either the mRNA-1273 vaccine from Moderna, the BNT162b2 vaccine from Pfizer-BioNTech, or the JNJ-78436735 vaccine from Janssen Pharmaceuticals. Participants were considered vaccinated if they received at least 2 doses of mRNA-1273 or BNT162b2 or 1 dose of JNJ-78436735 at least 14 days before the specimen collection date. Participants never vaccinated as of the specimen collection date were considered unvaccinated. Participants who were partially vaccinated, had documentation of more than 1 vaccine type, or had documentation of 3 or more vaccine doses were excluded from the study.

### Statistical Analysis

The crude vaccine effectiveness was defined as 1 minus the odds of being vaccinated among case individuals compared with control individuals, multiplied by 100. Logistic regression was used to calculate vaccine effectiveness and 95% CIs. Multivariable logistic regression was used to calculate vaccine effectiveness and 95% CIs adjusted for age group (<20 years, sequential 5-year age groups for 20-49 years, and ≥50 years), sex, race and ethnicity (American Indian/Alaska Native, Asian/Pacific Islander, Black, Hispanic, White, other, or unknown), service (Air Force, Army, Coast Guard, Marine Corps, or Navy), and region. Race and ethnicity came from DOD personnel records and was self-designated by the military member; “other” was an option reported by the individual, and “unknown” included individuals who did not report race or ethnicity. Region was defined by a military member’s unit location at the specimen collection date and was categorized as South, Northeast, Midwest, or West based on US census regions.^31^ Analyses were conducted separately by vaccine type and outcome. The outcomes were defined as overall SARS-CoV-2 infections, asymptomatic infections, symptomatic infections, and infections among individuals hospitalized for any reason. Asymptomatic cases were defined as individuals with no medical encounters for COVID-like illness within 10 days before or after the specimen collection date and no symptoms reported in the DRSi. Symptomatic cases were defined as those with a medical encounter for COVID-like illness within 10 days before or after the specimen collection date or symptoms reported in the DRSi. All controls were included in the analyses for overall, symptomatic, and asymptomatic infections. The analysis of COVID-19 among hospitalizations was restricted to individuals (cases and controls) with a hospitalization (any diagnosis, not restricted to COVID-19) within 30 days before or after the specimen collection date or a DRSi record indicating the person was currently hospitalized.

To account for differences in the time since vaccination and specimen collection date, a secondary (ie, sensitivity) analysis was performed that restricted participants to those vaccinated within 14 to 119 days before the specimen collection date.^8^ The absolute difference in the adjusted vaccine effectiveness of the restricted analysis and the original analysis was calculated, and the correlation statistic between these 2 estimates was then used to calculate the 95% CIs for the absolute differences. SAS, version 9.4 (SAS Institute) was used for all statistical analyses.

## Results

### Characteristics of the Study Population

From January 1 to September 24, 2021, there were 441 379 military personnel tested for a SARS-CoV-2 infection who met the study inclusion criteria. Of those, 290 256 (66%; 236 555 [81%] male; median age, 25 years [range, 17-68 years]) were tested during the pre-Delta period and 151 123 (34%; 120 536 [80%] male; median age, 26 years [range, 17-70 years]) during the Delta period. During the pre-Delta period, 38 968 participants (13%) were positive for SARS-CoV-2 and 251 288 (87%) tested negative. During the Delta period, 26 087 participants (17%) were positive for SARS-CoV-2, and 125 036 (83%) tested negative. Demographic characteristics of sex and race and ethnicity were similar between the cases and controls in the pre-Delta, Delta, and overall military populations (Table 1). However, the pre-Delta population was younger (≤24 years) (pre-Delta: 51% [cases] and 48% [controls]; Delta: 40% [cases and controls]; military population: 39%) and had a higher percentage of Army personnel (pre-Delta: 48% [cases] and 42% [controls]; Delta: 37% [cases] and 42% [controls]; military population: 35%) compared with the Delta and overall military population. Compared with controls, cases also had a higher percentage of personnel from the South (64% [pre-Delta and Delta] vs 60% [pre-Delta] and 56% [Delta]) and a lower percentage from the West (26% [pre-Delta] and 30% [Delta] vs 30% [pre-Delta] and 35% [Delta]).

**Table 1.** Demographic Characteristics of Military Members Who Tested Positive (Cases) and Negative (Controls) for SARS-CoV-2 Before and After the Predominance of the SARS-CoV-2 Delta Variant

Characteristics	Military personnel tested, No. (%)				
	Pre-Delta period		Delta period		Active component population (n = 1 220 721)
	Cases (n = 38 968)	Controls (n = 251 288)	Cases (n = 26 087)	Controls (n = 125 036)	
Sex					
Female	6853 (18)	46 848 (19)	5038 (19)	25 549 (20)	210 120 (17)
Male	32 115 (82)	204 440 (81)	21 049 (81)	99 487 (80)	1 010 601 (83)
Age category, y					
<20	4173 (11)	26 463 (11)	1369 (5)	9299 (7)	88 649 (7)
20-24	15 691 (40)	92 027 (37)	9114 (35)	40 949 (33)	389 887 (32)
25-29	8906 (23)	56 436 (22)	6345 (24)	29 317 (23)	281 353 (23)
30-34	5035 (13)	34 923 (14)	4114 (16)	19 670 (16)	194 481 (16)
35-39	3113 (8)	23 521 (9)	3070 (12)	14 561 (12)	147 328 (12)
40-44	1395 (4)	11 067 (4)	1422 (5)	7009 (6)	74 958 (6)
45-49	484 (1)	4512 (2)	467 (2)	2841 (2)	30 216 (2)
≥50	171 (0)	2339 (1)	186 (1)	1390 (1)	13 849 (1)
Race and ethnicity					
American Indian/Alaska Native	356 (1)	2252 (1)	217 (1)	1034 (1)	10 716 (1)
Asian/Pacific Islander	1609 (4)	10 532 (4)	807 (3)	5206 (4)	50 703 (4)
Black	7193 (18)	42 383 (17)	4986 (19)	20 247 (16)	190 148 (16)
Hispanic	7974 (20)	44 678 (18)	4822 (18)	21 844 (17)	210 295 (17)
White	19 971 (51)	137 435 (55)	13 806 (53)	69 304 (55)	681 513 (56)
Other or unknown^a^	1865 (5)	14 008 (6)	1449 (6)	7401 (6)	77 346 (6)
Service					
Air Force	7788 (20)	57 240 (23)	6979 (27)	32 050 (26)	277 644 (23)
Army	18 666 (48)	105 770 (42)	9640 (37)	51 980 (42)	432 041 (35)
Coast Guard	239 (1)	1460 (1)	268 (1)	1331 (1)	39 150 (3)
Marine Corps	4851 (12)	32 362 (13)	3984 (15)	14 701 (12)	163 559 (13)
Navy	7424 (19)	54 456 (22)	5216 (20)	24 974 (20)	308 327 (25)
Region					
Midwest	3164 (8)	17 418 (7)	1354 (5)	9668 (8)	90 041 (7)
Northeast	925 (2)	6909 (3)	161 (1)	2196 (2)	47 914 (4)
South	24 863 (64)	151 457 (60)	16 712 (64)	69 489 (56)	679 204 (56)
West	10 016 (26)	75 504 (30)	7860 (30)	43 683 (35)	403 562 (33)

^a^ Some individuals self-reported their race and ethnicity as other. For those who did not self-report a race or ethnicity, it was categorized as unknown.

During the pre-Delta period, only 466 cases (1%) were vaccinated, whereas 34 243 controls (14%) were vaccinated (Table 2). However, during the Delta period, 10 041 cases (38%) were vaccinated, whereas 81 486 controls (65%) were vaccinated (Table 2). Most participants in both periods received the BNT162b2 vaccine (64%-82%, depending on the population). The mRNA-1273 vaccine was the next most used vaccine (13%-32%), followed by the JNJ-78436735 vaccine (0%-8%).

**Table 2.** Vaccine Effectiveness During the Pre-Delta Period and the Delta Period by Outcome and Vaccine Type

Outcome, vaccine type	Pre-Delta period			Delta period		
	Military personnel, No.		Adjusted vaccine effectiveness, % (95% CI)^a^	Military personnel, No.		Adjusted vaccine effectiveness, % (95% CI)^a^
	Cases (n = 38 968)	Controls (n = 251 288)		Cases (n = 26 087)	Controls (n = 125 036)	
Unvaccinated	38 501	217 032	NA	16 044	43 537	NA
All SARS-CoV-2 infections						
Any vaccine	466	34 243	89.2 (88.1-90.1)	10 041	81 486	70.2 (69.3-71.1)
mRNA-1273	87	10 945	93.5 (91.9-94.7)	2011	24 259	79.4 (78.3-80.4)
BNT162b2	346	21 855	87.6 (86.2-88.9)	6480	50 943	69.3 (68.2-70.3)
JNJ-78436735	33	1443	81.8 (74.2-87.1)	1550	6284	38.3 (34.5-41.9)
Asymptomatic infections						
Any vaccine	159	34 243	85.5 (82.9-87.6)	3823	81 486	65.9 (64.3-67.5)
mRNA-1273	21	10 945	94.7 (91.9-96.6)	779	24 259	77.0 (75.1-78.8)
BNT162b2	130	21 855	80.3 (76.5-83.5)	2377	50 943	66.0 (64.0-67.8)
JNJ-78436735	8	1443	81.4 (62.6-90.8)	667	6284	19.6 (12.2-26.4)
Symptomatic infections						
Any vaccine	307	34 243	90.5 (89.3-91.5)	6218	81 486	72.3 (71.3-73.3)
mRNA-1273	66	10 945	93.1 (91.2-94.6)	1232	24 259	80.6 (79.4-81.8)
BNT162b2	216	21 855	89.9 (88.4-91.2)	4103	50 943	71.0 (69.7-72.1)
JNJ-78436735	25	1443	82.4 (73.9-88.2)	883	6284	48.9 (45.0-52.7)
Hospitalizations						
Unvaccinated	616	6198	NA	214	1254	NA
Any vaccine	10	933	88.6 (78.2-94.0)	43	1209	86.4 (80.5-90.5)
mRNA-1273	2	199	89.6 (57.5-97.4)	9	249	88.1 (75.7-94.2)
BNT162b2	8	711	88.0 (75.4-94.1)	27	893	88.4 (82.1-92.5)
JNJ-78436735	0	23	NA	7	67	57.7 (2.6-81.6)

Abbreviation: NA, not applicable.

^a^ The analysis of vaccine effectiveness was adjusted for age group, sex, race and ethnicity, service (Air Force, Army, Coast Guard, Marine Corps, or Navy), and US census region.

Most cases in the pre-Delta (66%) and Delta (62%) periods were symptomatic. However, only a small percentage of cases resulted in hospitalizations (pre-Delta: 2%; Delta: 0.4%). This distribution was consistent across all vaccine types.

### Vaccine Effectiveness

#### All SARS-CoV-2 Infections

Vaccine effectiveness against all SARS-CoV-2 infections for any vaccine type during the pre-Delta period was 89.2% (95% CI, 88.1%-90.1%) (Table 2). During the Delta period, the overall effectiveness was reduced by 19%, resulting in a significantly lower estimate of 70.2% (95% CI, 69.3%-71.1%). When stratified by vaccine type, the lower effectiveness during the Delta period compared with the pre-Delta period remained. Effectiveness was greatest among recipients of the mRNA-1273 vaccine in the pre-Delta (93.5%; 95% CI, 91.9%-94.7%) and Delta (79.4%; 95% CI, 78.3%-80.4%) periods. The JNJ-78436735 vaccine had the lowest effectiveness of the 3 vaccines during the pre-Delta (81.8%; 95% CI, 74.2%- 87.1%) and Delta (38.3%; 95% CI, 34.5%-41.9%) periods and the largest decrease in effectiveness between the 2 periods.

#### Asymptomatic Infections

The lowest vaccine effectiveness was seen for asymptomatic infections compared with symptomatic infections and hospitalizations with the exception of the mRNA-1273 vaccine during the pre-Delta period, although statistical significance varied by vaccine type (Table 2). The mRNA-1273 vaccine had the highest effectiveness of the 3 vaccines for asymptomatic infections at 94.7% (95% CI, 91.9%-96.6%) in the pre-Delta period and 77.0% (95% CI, 75.1%-78.8%) in the Delta period. The BNT162b2 and JNJ-78436735 vaccines had similar effectiveness against asymptomatic infections during the pre-Delta period (BNT162b2: 80.3% [95% CI, 76.5%-83.5%]; JNJ-78436735: 81.4% [95% CI, 62.6%-90.8%]), but the effectiveness decreased substantially for the JNJ-78436735 vaccine during the Delta period (19.6%; 95% CI, 12.2%-26.4%). The absolute reduction in effectiveness for asymptomatic infections from the pre-Delta to Delta period for the mRNA-1273, BNT162b2, and JNJ-78436735 vaccines was 17.7%, 14.3%, and 61.8%, respectively.

#### Symptomatic Infections

The effectiveness of all 3 vaccines against symptomatic infection was high during the pre-Delta period but decreased during the Delta period (Table 2). Similar to asymptomatic infections, effectiveness was highest for the mRNA-1273 and BNT162b2 vaccines in the pre-Delta period (mRNA-1273: 93.1% [95% CI, 91.2%-94.6%]; BNT162b2: 89.9% [95% CI, 88.4%-91.2%) and the Delta period (mRNA-1273: 80.6% [95% CI, 79.4%-81.8%]; BNT162b2: 71.0% [95% CI, 69.7%-72.1%]). However, for the JNJ-78436735 vaccine, effectiveness decreased from 82.4% (95% CI, 73.9%-88.2%) in the pre-Delta period to 48.9% (95% CI, 45.0%-52.7%) in the Delta period. The absolute reduction in effectiveness for symptomatic infections from the pre-Delta to Delta period for the mRNA-1273, BNT162b2, and JNJ-78436735 vaccines was 12.5%, 18.9%, and 33.5%, respectively.

#### Hospitalizations

Vaccine effectiveness among hospitalized individuals was limited by a smaller sample size, with only 10 cases in the pre-Delta period and 43 cases in the Delta period (Table 2). Vaccine effectiveness point estimates were statistically significant for both periods, but a comparison could not be made between the pre-Delta and Delta periods. From the pre-Delta to Delta periods, the mRNA-1273 vaccine effectiveness decreased from 89.6% (95% CI, 57.5%-97.4%) to 88.1% (95% CI, 75.7%-94.2%) and the BNT162b2 vaccine effectiveness decreased from 88.0% (95% CI, 75.4%-94.1%) to 88.4% (95% CI, 82.1%-92.5%). The change in effectiveness for hospitalized individuals from the pre-Delta to the Delta period was a decrease of 1.5% for the mRNA-1273 vaccine and an increase of 0.4% for the BNT162b2 vaccine. There were no hospitalized cases among JNJ-78436735 recipients during the pre-Delta period; thus, the crude effectiveness was 100% but the adjusted effectiveness could not be calculated. During the Delta period, however, the effectiveness among hospitalized individuals vaccinated with JNJ-78436735 was 57.7% (95% CI, 2.6%-81.6%).

### Vaccine Effectiveness Within 14 to 119 Days of Vaccination

Results of the secondary analysis restricting the time from vaccination to specimen collection to 14 to 119 days, compared with the original analysis, are shown in Table 3. The time restriction did not substantially affect vaccine effectiveness estimates during the pre-Delta period because most participants (99.8%) had their specimen collected within this time frame. For the Delta period, 46% to 72% (depending on vaccine type) of participants had their specimen collected within 14 to 119 days after completion of their vaccine series. The time restriction for the Delta period resulted in vaccine effectiveness estimates that were a mean absolute difference of 4.8 percentage points (range, 0.8-11.7 percentage points, depending on vaccine type and outcome) higher than the original analysis estimates.

**Table 3.** Comparison of Original and Restricted Analyses for SARS-CoV-2 Vaccine Effectiveness During the Pre-Delta and Delta Periods by Outcome and Vaccine Type

Outcome, vaccine type	Adjusted vaccine effectiveness estimate (95% CI)^a^		Absolute difference (95% CI)
	Restricted analysis^b^	Original analysis^c^	
**Delta**			
All SARS-CoV-2 infections			
Any vaccine	74.6 (73.6 to 75.6)	70.2 (69.3 to 71.1)	4.4 (4.2 to 4.6)
mRNA-1273	85.0 (83.7 to 86.2)	79.4 (78.3 to 80.4)	5.6 (5.3 to 5.9)
BNT162b2	74.9 (73.7 to 76.0)	69.3 (68.2 to 70.3)	5.6 (5.4 to 5.8)
JNJ-78436735	45.1 (40.7 to 49.1)	38.3 (34.5 to 41.9)	6.8 (6.1 to 7.5)
Asymptomatic infections			
Any vaccine	70.2 (68.3 to 71.9)	65.9 (64.3 to 67.5)	4.3 (4.0 to 4.6)
mRNA-1273	82.8 (80.3 to 84.9)	77.0 (75.1 to 78.8)	5.8 (5.3 to 6.3)
BNT162b2	70.6 (68.3 to 72.7)	66.0 (64.0 to 67.8)	4.6 (4.2 to 5.0)
JNJ-78436735	31.3 (22.8 to 38.9)	19.6 (12.2 to 26.4)	11.7 (10.3 to 13.1)
Symptomatic infections			
Any vaccine	76.8 (75.7 to 77.9)	72.3 (71.3 to 73.3)	4.5 (4.3 to 4.7)
mRNA-1273	86.1 (84.6 to 87.5)	80.6 (79.4 to 81.8)	5.5 (5.2 to 5.8)
BNT162b2	77.0 (75.7 to 78.3)	71.0 (69.7 to 72.1)	6.0 (5.8 to 6.2)
JNJ-78436735	52.2 (47.5 to 56.6)	48.9 (45.0 to 52.7)	3.3 (2.4 to 4.2)
Hospitalizations			
Any vaccine	87.9 (81.0 to 92.3)	86.4 (80.5 to 90.5)	1.5 (0.5 to 2.5)
mRNA-1273	92.8 (76.6 to 97.8)	88.1 (75.7 to 94.2)	4.7 (2.8 to 6.6)
BNT162b2	89.2 (81.5 to 93.7)	88.4 (82.1 to 92.5)	0.8 (−0.4 to 2.0)
JNJ-78436735	59.5 (−6.3 to 84.6)	57.7 (2.6 to 81.6)	1.8 (−6.6 to 10.2)
**Pre-Delta**			
All COVID-19 cases			
Any vaccine	89.1 (88.1 to 90.1)	89.2 (88.1 to 90.1)	−0.1 (−0.2 to 0.0)
mRNA-1273	93.4 (91.9 to 94.7)	93.5 (91.9 to 94.7)	−0.1 (−0.3 to 0.1)
BNT162b2	87.5 (86.1 to 88.8)	87.6 (86.2 to 88.9)	−0.1 (−0.3 to 0.1)
JNJ-78436735	82.8 (75.4 to 87.9)	81.8 (74.2 to 87.1)	1.0 (0.1 to 1.9)
Asymptomatic infections			
Any vaccine	85.3 (82.7 to 87.5)	85.5 (82.9 to 87.6)	−0.2 (−0.5 to 0.1)
mRNA-1273	94.7 (91.8 to 96.6)	94.7 (91.9 to 96.6)	0.0 (−0.3 to 0.3)
BNT162b2	80.0 (76.1 to 83.2)	80.3 (76.5 to 83.5)	−0.3 (−0.8 to 0.2)
JNJ-78436735	81.2 (62.3 to 90.7)	81.4 (62.6 to 90.8)	−0.2 (−2.2 to 1.8)
Symptomatic infections			
Any vaccine	90.5 (89.4 to 91.6)	90.5 (89.3 to 91.5)	0.0 (−0.2 to 0.2)
mRNA-1273	93.1 (91.2 to 94.6)	93.1 (91.2 to 94.6)	0.0 (−0.2 to 0.2)
BNT162b2	89.9 (88.4 to 91.2)	89.9 (88.4 to 91.2)	0.0 (−0.2 to 0.2)
JNJ-78436735	83.8 (75.4 to 89.2)	82.4 (73.9 to 88.2)	1.4 (0.4 to 2.4)
Hospitalizations			
Any vaccine	88.3 (77.8 to 93.9)	88.6 (78.2 to 94.0)	−0.3 (−1.4 to 0.8)
mRNA-1273	89.6 (57.5 to 97.4)	89.6 (57.5 to 97.4)	0.0 (−2.8 to 2.8)
BNT162b2	87.6 (74.7 to 93.9)	88.0 (75.4 to 94.1)	−0.4 (−1.8 to 1.0)
JNJ-78436735	NA	NA	NA

Abbreviation: NA, not applicable.

^a^ The analysis of vaccine effectiveness was adjusted for age group, sex, race and ethnicity, service (Air Force, Army, Coast Guard, Marine Corps, or Navy), and US census region.

^b^ Restricted to participants vaccinated within 14 to 119 days before the specimen collection date.

^c^ No limits on time since vaccination.

## Discussion

Although there are several published studies on the effectiveness of mRNA COVID-19 vaccines^5,6,7,8,9,10,11,12,13,14,15,16,17,18,19,20,21,22^ and a few conducted during the period when the Delta variant predominated,^6,8,32,33,34^ there is a lack of published data on the effectiveness of the JNJ-78436735 vaccine. This is one of the first studies to evaluate the association of JNJ-78436735 vaccination with infections and hospitalizations, especially during the period when the Delta variant predominated. In this study, before the predominance of the Delta variant, the effectiveness of the JNJ-78436735 vaccine against SARS-CoV-2 infections was high and similar to that of the mRNA vaccines. However, during the Delta period, the effectiveness of the JNJ-78436735 vaccine decreased significantly and with greater magnitude than did that of the mRNA-based vaccines. From the pre-Delta to the Delta period, the absolute decrease in effectiveness of the JNJ-78436735 vaccine was 61.8 and 33.5 percentage points for asymptomatic and symptomatic infections, respectively, suggesting that JNJ-78436735 vaccination was associated with only moderate protection against symptomatic and minimal protection against asymptomatic infections. The vaccine was associated with moderate protection against hospitalizations, but the estimate was significantly lower than that for the 2 mRNA-based vaccines. Only a few identified studies have evaluated the JNJ-78436735 vaccine, and they found a similar decrease in effectiveness during the Delta period.^19,20,21,22^ These results suggest that among US military personnel, JNJ-78436735 vaccination was associated with some protection during the time of the SARS-CoV-2 Delta variant predominance but effectiveness was suboptimal compared with those of mRNA-based vaccines; this study’s results support use of a booster or second vaccine dose to increase effectiveness.

For the mRNA-based COVID-19 vaccines, we found a significant decrease in effectiveness against symptomatic and asymptomatic SARS-CoV-2 infections during the Delta period compared with the pre-Delta period. An overall absolute reduction of 12.5% to 18.9% in vaccine effectiveness against SARS-CoV-2 infections in nonhospitalized individuals was seen for the mRNA-based vaccines. This is similar to a report among the general population in England, which found a 12% absolute decrease in vaccine effectiveness in the Delta period compared with the pre-Delta period for the BNT162b2 vaccine.^5^ Similarly, among health care workers in the US, 2 other studies reported 14% to 25% absolute decreases in effectiveness of mRNA-based vaccines from the pre-Delta to Delta periods.^6,8^ However, among hospitalized individuals in the current study, vaccine effectiveness remained high during the Delta period and had only a 1.5% decrease for the mRNA-1273 vaccine and 0.4% increase for the BNT162b2 vaccine from the pre-Delta to the Delta period. Three recent studies among adolescents, a population that may be similar to military personnel, also found high effectiveness (91%-93%) of the BNT162b2 vaccine during the Delta period.^32,33,34^ Taken together, these findings suggest that although the effectiveness of mRNA-based COVID-19 vaccines was lower during the Delta period, these vaccines still were associated with relatively high protection against infection, especially against more severe outcomes.

The decrease in vaccine effectiveness during the Delta period could partially be explained by time since vaccination, indicating waning immunity, which was reported in a study by Tartof et al^6^ among health care workers. However, in the current study, after conducting the secondary (sensitivity) analysis evaluating vaccination within a 4-month window, the results still showed a decrease in the effectiveness of all 3 vaccines against asymptomatic and symptomatic infections during the Delta period. Effectiveness estimates during the Delta period were a mean 4.8 percentage points lower with this restriction. This is consistent with the findings of a study by Fowlkes et al,^8^ which also found only a 5–percentage point increase in effectiveness within a 4-month window from vaccination and a 7–percentage point decrease in effectiveness with a window greater than 5 months among frontline workers. Another study, by Fraley et al,^7^ found that waning immunity was less prominent in individuals aged 18 to 49 years compared with those 50 years or older. Because 99% of the population in the current study was less than 50 years of age, our findings are consistent with those of the study by Fraley et al. Therefore, although waning immunity occurred as time since vaccination increased, this study’s results support a decrease in vaccine effectiveness during the time of Delta variant predominance for these 3 COVID-19 vaccines.^35,36^

### Limitations

This study has limitations. It was restricted to the US military population in the continental US, which is younger and healthier than the general US population. Therefore, these findings are not generalizable to the entire US population. Because this population is generally healthy and high-risk COVID-19 medical conditions in this population are relatively rare, we did not adjust for comorbidities.^37^ If there were unexpected differences in the prevalence of COVID-19 high-risk conditions among cases and controls, they may have confounded the estimates. The analysis may also have been confounded by differences in risk of infection by vaccination status. Initial vaccination strategies focused on high-risk personnel, such as health care workers, but as vaccine availability increased, all military personnel were eligible for vaccination.^23^ Differences in risk would be most pronounced in the pre-Delta period and would have biased the results toward the null. Department of Defense vaccine mandates did not go into effect until late in the study period. Completeness of vaccine records could also have biased the results toward the null if individuals were misclassified as unvaccinated. However, because the data were collected after the DOD vaccine mandate was enforced and COVID-19 vaccination became a tracked force-readiness measure, vaccination records should have been mostly complete.

In addition, there may have been misclassification of symptomatic and asymptomatic cases owing to the use of administrative medical databases and a lack of medical encounters for some SARS-CoV-2 tests. The DRSi data, which require entry by physicians and public health personnel and include symptoms and hospitalization reporting, were used to lessen the possibility of misclassification. In all, 77.6% of the SARS-CoV-2 laboratory records had a DRSi record.

An additional limitation of the study is the use of time to define vaccine effectiveness against the Delta variant. This may have led to some misclassification of cases as infected with the Delta variant vs other SARS-CoV-2 variants. Currently, the SARS-CoV-2 sequencing data for the DOD is performed in a deidentified manner and cannot be linked to other data, necessitating the need to use time as a proxy. However, the use of the washout period and Centers for Disease Control and Prevention surveillance data should have minimized this effect.^29^ There was also an inability to completely separate the associations of the Delta period and waning immunity with vaccine effectiveness. Although the sensitivity analysis attempted to control for waning immunity, the 4-month window may have been too wide and could have minimized the association of waning immunity with vaccine effectiveness estimates.

## Conclusions

In the case-control study, the effectiveness of 3 COVID-19 vaccines against SARS-CoV-2 infection decreased during the time of the SARS-CoV-2 Delta variant predominance in the US among US military personnel. The decrease was significantly more pronounced among recipients of the JNJ-78436735 vaccine compared with the mRNA vaccines, even among hospitalized individuals. However, these findings should be interpreted with caution owing to the multiple limitations and unmeasured confounders. As new variants of the SARS-CoV-2 virus emerge, such as the Delta and Omicron variants, and as time since initial vaccination increases, these finding suggest a continued need to evaluate the effectiveness of the vaccines and booster doses and to explore new vaccine formulations to maintain protection against SARS-CoV-2 infection.

## Notes

Supplement. **eAppendix.** *International Statistical Classification of Diseases, Tenth Revision, Clinical Modification (ICD-10-CM)* Codes for COVID-like Illness Case Definition
