# Supplementary material for: Effectiveness of mRNA-1273, BNT162b2, and JNJ-78436735 COVID-19 Vaccines Among US Military Personnel Before and During the Predominance of the Delta Variant
Source: JAMA Netw Open. 2022 Apr 20;5(4):e228071. doi: 10.1001/jamanetworkopen.2022.8071 (PMC9021911; doi:10.1001/jamanetworkopen.2022.8071)
Supplement: Supplement. — eAppendix. International Statistical Classification of Diseases, Tenth Revision, Clinical Modification (ICD-10-CM) Codes for COVID-like Illness Case Definition [file jamanetwopen-e228071-s001.pdf]

Supplementary Online Content

Eick-Cost AA, Ying S, Wells N. Effectiveness of mRNA-1273, BNT162b2, and JNJ-78436735 COVID-19 vaccines among US military personnel before and during the predominance of the Delta variant. *JAMA Netw Open*. 2022;5(4):e228071. doi:10.1001/jamanetworkopen.2022.8071

eAppendix. *International Statistical Classification of Diseases, Tenth Revision, Clinical Modification (ICD-10-CM)* Codes for COVID-like Illness Case Definition

This supplementary material has been provided by the authors to give readers additional information about their work.

© 2022 Eick-Cost AA et al. *JAMA Network Open*.

**eAppendix.** ***International Statistical Classification of Diseases, Tenth Revision, Clinical Modification (ICD-10-CM)*** **Codes for COVID--like Illness Case Definition**	
**ICD-10-CM Code**	**Description**
B34.2	Coronavirus, unspecified
B97.21	SARS-associated coronavirus as the cause of disease classified elsewhere
B97.29	Other coronavirus as the cause of diseases classified elsewhere
J00	Acute nasopharyngitis; common cold
J06.9	Acute upper respiratory infection, unspecified
J12.81	Pneumonia due to SARS-associated coronavirus
J12.89	Other viral pneumonia
J12.9	Viral pneumonia unspecified
J16.8	Pneumonia due to other specified infectious organism
J17	Pneumonia in diseases classified elsewhere
J18.0	Bronchopneumonia, unspecified organism
J18.1	Lobar pneumonia, unspecified organism
J18.8	Other pneumonia, unspecified organism
J18.9	Pneumonia, unspecified organism
J20.8	Acute bronchitis due to other specified organisms
J20.9	Acute bronchitis, unspecified
J22	Unspecified acute lower respiratory infection
J40	Bronchitis, not specified as acute or chronic
J80	Acute Respiratory Distress Syndrome
J84.111	Idiopathic interstitial pneumonia not otherwise specified
R05	Cough
R06.0	Dyspnea
R06.00	Dyspnea, unspecified
R06.02	Shortness of Breath
R06.03	Acute Respiratory Distress
R06.09	Other forms of dyspnea
R43.0	Anosmia
R43.2	Ageusia
R50.9	Fever, unspecified
U07.1	2019-nCoV acute respiratory disease, COVID-19, virus identified

© 2022 Eick-Cost AA et al. *JAMA Network Open*.
